# Did weekly economic index and volatility index impact US food sales during the first year of the pandemic?

**DOI:** 10.1186/s40854-023-00460-y

**Published:** 2023-02-10

**Authors:** Narasingha Das, Partha Gangopadhyay

**Affiliations:** 1 Economists for Peace and Security- Australia Chapter, Sydney, Australia; 2 Western Sydney University, Sydney, Australia

**Keywords:** COVID-19, Food sales, US weekly economic index, CBOE’s volatility index, ARDL model, Bewley transformation, NARDL model, QARDL model, E32, E00, Q54, C33

## Abstract

We explore the impacts of economic and financial dislocations caused by COVID-19 pandemic shocks on food sales in the United States from January 2020 to January 2021. We use the US weekly economic index (*WEI*) to measure economic dislocations and the Chicago Board Options Exchange volatility index (*VIX*) to capture the broader stock market dislocations. We validate the NARDL model by testing a battery of models using the autoregressive distributed lags (ARDL) methodology (ARDL, NARDL, and QARDL specifications). Our study postulates that an increase in *WEI* has a significant negative long-term effect on food sales, whereas a decrease in *WEI* has no statistically significant (long-run) effect. Thus, policy responses that ignore asymmetric effects and hidden cointegration may fail to promote food security during pandemics.

## Introduction

The COVID-19 pandemic (hereafter pandemic) has caused an unprecedented global health crisis, causing millions of people to become ill or die. In addition, the pandemic caused unprecedented global shocks to the regional and global economic systems. The magnitude of the economic shocks stems from globally synchronized lockdowns and serious financial system dislocations (see Apergis and Apergis 2021). Although the full effect of the pandemic is still unclear, our paper explores the effect of mitigation strategies (e.g., lockdowns and social distancing) and financial system shocks on the US food sales. Food sales are critical for ensuring food security. A better understanding is required to assess whether the pandemic has had a significant impact on the world’s food supply chains (see World Bank 2020; IFPRI 2020), which can have a negative long-term effect on health due to insufficient nutrient intakes even in developed nations. According to recent research, economic disruptions resulted in lost income, which is a serious implication for food sales and security (Singh et al. 2020). Economic disruptions are known to have triggered falling food sales and rising hunger and malnutrition in low-income countries, with no known effects on food sales in advanced economies (see Mogues 2020).

This study focuses on one of the most advanced economies, the United States, which was severely impacted by the COVID-19 pandemic. We want to understand whether economic disruptions from the first wave of pandemic and shocks to financial markets, particularly the equity market, have compromised (short-run or long-run) food sales in the United States.^1^ To avoid misunderstanding, we explain the rationale for conserving food sales as a variable in the United States: in the United States, the primary public food assistance program, Supplemental Nutrition Assistance Program (SNAP), seeks to protect the poor from food entitlement failures. The SNAP operates as an electronic benefit transfer that low-income people can use at retail food establishments. Hence, there is little reason to believe that the pandemic has increased food insecurity among the poor (those earning less than a certain level of income) who are covered by the public food assistance program. Similarly, there is an income threshold above in which food insecurity makes little sense since these people have the purchasing power to buy the required bundle of food items. However, the pandemic may have impacted food sales to those with intermediate incomes between these two income thresholds (lower and upper). Changes in retail food sales data can provide insight into the dynamics of food consumption of these people whose incomes are higher than the SNAP level but lower than the level that accords the full purchasing power for buying the desired bundles of food items, regardless of prices and/or economic and financial disruptions.

Several authors, including Apergis and Apergis (2021), have argued that the pandemic has impacted financial markets (primarily stock and commodity markets), sending shockwaves throughout the economy.

We developed an autoregressive distributed lags (ARDL) model to capture both the short-run and long-run effects of the weekly economic index (*WEI*) and the weekly volatility index (*VIX*) on (weekly) food sales or food consumption (*Z*_1_).^2^ To capture hidden cointegration and asymmetric effects of economic and financial shocks on food sales, we then apply the nonlinear ARDL (NARDL) methodology.^3^ We further extend the analysis to determine whether the relationship between food sales and *WEI* and *VIX* varies across quantiles of the conditional distribution of food sales. For this purpose, we apply the QARDL model of Cho et al. (2015) to uncover previously unknown aspects of the quantile-specific relationships between food sales and the economic consequences of the pandemic.^4^ We use the Bewley transformation as a robustness check to test whether endogeneity issues have compromised the estimation.^5^

The remainder of this paper is as follows. Section "Background literature and empirical strategy" provides a brief review of the emerging literature and discusses the econometric methodology. Section "Results and discussion" discusses the empirical findings and their implications. Finally, Section "Conclusion" concludes the paper.

## Background literature and empirical strategy

COVID-19 has caused severe supply shocks, similar to the effects of natural disasters on supply chains. However, it has been found that richer nations are more resilient to shocks than poorer nations. Our research context is one of the world’s wealthiest countries, the United States, which was also severely impacted by pandemic-related disruptions.

### Economic disruptions

Despite a series of severe economic disruptions, the US economy has several layers of protection in place to deal with the mounting challenges of the pandemic’s economic and financial disruptions: First, one of the implicit insurances against the spillover effect of these disruptions to food consumption (or, food sales) is the ability of the US economy to resume economic growth, as highlighted in the context of US natural disasters by Anbarci et al. (2005) and confirmed by Kellenberg and Mobarak (2008). Second, as Kahn (2005) and Toya and Skidmore (2007) discovered, the quality of institutions provides an effective barrier to the spread of disruptions and shocks throughout the economy. Third, with effective policy interventions, the short-term adverse effects of such disruptions can be avoided from becoming long-term problems. However, it is also understood that recovery is not always guaranteed (see Noy 2009; Cavallo et al. 2013). The present study introduces the US *WEI* as a proxy for economic disruptions caused by the pandemic. In the context of the COVID-19 pandemic, several papers have used *WEI* to track the economic disruptions caused by the pandemic. Some of the key papers are Aprigliano et al. (2022), Ashraf (2020), Baumeister et al. (2022), Carriero et al. (2022), Koop et al. (2022), and Lewis et al. (2021a, 2021b).

Similarly, as we will see in the following subsection, we use the Chicago Board Options Exchange (CBOE) *VIX* as a proxy for financial market disruptions. Our research is based on examining the relationship between food sales and the *VIX* and *WEI*.

### Financial disruptions and roles of non-fundamentals

Besides economic disruptions, pandemic-related news and reports spread people’s panic and increase investor anxiety. The fear of investors had a significant impact on commodity prices (Atri et al. 2021). Since the 2008 financial crisis, the impacts of non-fundamentals, such as participants’ expectations, anxiety, and market sentiments, on financial markets have been thoroughly examined (see Joo et al. 2020). Baker et al. (2016) also emphasized the role of policy uncertainty in influencing participants’ beliefs, macroeconomic activities, and thus financial markets (Aloui et al. 2016). Therefore, the increasing anxiety among investors in financial markets, resulted in rising “investor fear,” which is measured by the CBOE *VIX*. COVID-19’s impact on food sales can be seen through its effect on *VIX*. This aspect has remained relatively unexplored despite its potentially devastating short- and long-term consequences for human security. We intend to fill this void by investigating potential nonlinear and asymmetric relationships between food sales and explanatory variables (*WEI* and *VIX*). It is critical to note that market sentiment can be measured using a volatility index derived from the GARCH model. Our results using the volatility index are roughly in conformity with CBOE indicator of *VIX*. Alternatively, the economic sentiment index developed by Shapiro et al. (2022) can be used to assess the news sentiment of the pandemic. Because our focus is not the news sentiment in this work, we choose not to use the economic sentiment index.

### COVID-19 and commodity markets

The following summarizes the effects of economic disruptions on commodity markets during the pandemic: First, the outbreak lowered most commodity prices, particularly crude oil. For example, oil prices tumbled by two-thirds in the first quarter of 2020, a never-before-seen phenomenon. Such decreases have a wide-ranging impact on the cost of production for most goods and thus on the supply side. However, the demand side also plummeted due to COVID-19 containment measures, which caused a drop in economic activity. Thus, the pandemic caused a unique combination of disruptions in commodity markets, affecting both demand and supply (see Baffes et al. 2020). According to Baffes et al. (2020), disruptions in the food supply chain may have impacted food sales concerns and hoarding by consumers and speculators.

Simultaneously, the pandemic disrupted agricultural commodity production due to shortages of key inputs, such as fertilizers, primarily due to mitigation measures. The labor movement across state and country borders was heavily regulated. Lockdowns also caused severe supply shocks in the food procuring industries. Moreover, trade restrictions exacerbated the food consumption problem (see Glauber et al. 2020; Schmidhuber et al. 2020; World Bank 2020).

### Variables and data

This study employs the *WEI*^6^ data for the United States and the CBOE *VIX* to model the short- and long-run effects of a pandemic from January 2020 to January 2021. The *WEI* is an index of real economic activity’s ten indicators that are scaled to correspond to the four-quarter GDP growth rate. It is a series component that covers consumer behavior, the labor market, and production (Lewis et al. 2021a, 2021b). Following the extant literature, we know that the supply and demand shocks caused by the COVID-19 pandemic have an impact on commodity markets, but the commodity market is also impounded by non-fundamentals such as participants’ expectations (as discussed in Joo et al. 2020; Baker et al. 2016; Aloui et al. 2016; Baumeister and Peersman 2013; Kilian 2009). The CBOE *VIX* is used to capture market expectations, whereas the US *WEI* is used to capture economic disruptions caused by the COVID-19 pandemic.

The CBOE *VIX* is a real-time market index issued by the CBOE as a volatility index to indicate the market’s expectations for the relative strengths of near-term price changes (over the next 30 days) (of the S&P 500 index (SPX)). Smales (2014) emphasized the importance of the *VIX* as a measure of investor fear. Thus, increases (decreases) in *VIX* indicate higher (lower) investor fear about the future. Hence, we use *VIX* as an indicator of the pandemic’s long-term (economic) consequences. It is critical to note that the long-term in the context of a pandemic can be a few weeks due to potential upheavals in economic and financial situations caused by the pandemic. Meanwhile, *WEI* captures the short-run impacts of economic disruption caused by the pandemic.

For the food sales data, we use a weekly series released by the US Department of Agriculture (USDA). The USDA publishes the weekly retail food sales series, which is derived from proprietary scanner data using a nationally representative sample of retail food establishments collected by Information Resources Inc. [see https://www.ers.usda.gov/data-products/weekly-retail-food-sales/]. The USDA also publishes a volume index of retail food sales. We alternately use these two series as dependent variables as indicators of food sales. The N/ARDL and QARDL methodologies are used to detect the effects of *VIX* and *WEI* on *Food Security* (*Z*_1_) in the United States.

### Empirical models

#### Baseline ARDL model

For assessing the impacts of *VIX* and *WEI*, we postulate an empirical relationship using the standard ARDL model. Equation (1) presents the standard ARDL (p, q) model:

1 $$\begin{aligned}LnZ_{1t} &= \alpha_{0} + \pi \, LnZ_{1t - 1} + \mathop \sum \limits_{i = 1}^{p} \alpha_{1i} \Delta LnVIX_{{\left( {t - 1} \right) - i}} + \mathop \sum \limits_{i = 0}^{q} \alpha_{2i} \Delta LnWEI_{t - i} \\&\quad+ \, \beta_{1} LnWEI_{t - 1} + \, \beta_{2} LnVIX_{{\left( {t - 1} \right) - 1}} + \, \varepsilon_{t} \end{aligned}$$

where *α*_0_ is the intercept term, π is the error correction term, *α*_1*i*_ & *α*_2*i*_ is the short-run coefficient for each variable while the parameter *β*_*1*_ & *β*_*2*_ is the long-run coefficient. Note that ε_t_ is the error term and *ln* is the natural logarithmic transformation. The ARDL bounds test enables us to model both I (0) and I (1) variables together. The null hypotheses H_0_ posits: *β*_*1*_ = *β*_*2*_ = 0. As a result, the null hypothesis assumes that there is no cointegration, whereas the alternative hypothesis assumes that there is cointegration. As a result, the alternative hypothesis H1 proposes that at least one parameter I is not zero. Using the Wald test, the F-statistics will be calculated to compare with the critical values of Pesaran et al. (2001). The ARDL mechanism detects cointegration if the calculated F-statistics are greater than the upper bound of critical values.

#### Nonlinear autoregressive distributed lag model: an extension

The evolving, albeit limited, literature on the pandemic’s effects *implicitly* suggests that the effects of increases and decreases in pandemic-related variables (on other variables) are *symmetric,* with increases and decreases assumed to work along the same functional relationship (see Ashraf 2020; Apergis and Apergis 2021; Bakry et al. 2021; Zaremba et al. 2020). In section  “Baseline ARDL model”, the ARDL model follows the path of the existing work. In this Section, we consider the possibility of asymmetric relationships between food sales (*Z*_1_) and the *WEI* and *VIX*. We contend that changes in the *WEI* and *VIX* can send specific messages to policymakers about the state of the economy and financial system, to which policymakers may respond asymmetrically. If there are any asymmetries, the symmetric models are incorrectly specified. As a result, we argue that a nonlinear and asymmetric error correction model based on a collection of (nonlinear autoregressive distributed lag) NARDL models will better capture the dependent variable’s responses to various shocks in the independent variables of interest when these shocks have underlying asymmetric effects.

To avoid the ARDL model’s potential technical inconsistencies, the analysis is performed using the NARDL approach described above. The NARDL model developed by Greenwood-Nimmo and Shin (2013) and Shin et al. (2014) allows us to examine the short-run and long-run responses of Food sales (*Z*_1_) to asymmetric fluctuations in *VIX*, *WEI*. Furthermore, the NARDL model detects any hidden cointegration that other cointegration models fail to detect. Following the study’s motivation, the following nonlinear and asymmetric NARDL model (ignoring short-run dynamics) was applied to the US economy:

2a $$Z_{1t} = \beta^{ + } WEI_{t - 1}^{ + } + \beta^{ - } WEI_{t - 1}^{ - } + \theta^{ + } VIX_{{\left( {t - 1} \right) - 1}}^{ + } + \theta^{ - } VIX_{{\left( {t - 1} \right) - 1}}^{ - } { } + \mu_{t}$$

where Z_1_ is the chosen measure of food sales, $WEI$ is the US weekly economic index, $\beta^{ + }$ is the long-run coefficient associated with an increase in $WEI$, $WEI_{t - 1}^{ + }$, which coveys a message of further disruptions of the economy in our model. Similarly, $WEI_{t - 1}^{ - }$ conveys the message that (mitigation) disruptions are likely to decrease. This method divides changes in an independent variable’s values into positive ( +) and negative (-) partial sums, as shown below:

$$\begin{aligned} & WEI_{t} = WEI_{0} + WEI_{t}^{ + } + WEI_{t}^{ - } { },\,{\text{such}}\,{\text{that}}\quad WEI_{t}^{ + } = \mathop \sum \limits_{i = 1}^{t} \Delta WEI_{i}^{ + } = \mathop \sum \limits_{i = 1}^{t} \max \left( {\Delta WEI_{i} ,0} \right) \\ & \quad {\text{and}}\,\,WEI_{t}^{ - } = \mathop \sum \limits_{i = 1}^{t} \Delta WEI_{i}^{ - } = \mathop \sum \limits_{i = 1}^{t} \min \left( {\Delta WEI_{i} ,0} \right). \\ \end{aligned}$$

Note, $\beta^{ - }$ is the long-run coefficient associated with the negative change in $WEI_{t - 1}$, $WEI_{t - 1}^{ - }$, which brings the message of decreases in future disruptions to the economy. Note that that $\beta^{ + }$ and $\beta^{ - }$ are the positive partial sum and the negative partial sum of the increases and decreases in $WEI_{t - 1}$, respectively. Similarly, we define the asymmetric change in $VIX$_(*t*−1)−1_ as a measure of financial market (investor) fear as follows: an increase in $VIX$_(*t*−1)−1_ carries a message about the rise in future uncertainty, whereas a decrease in $VIX$_(*t*−1)−1_ indicates a decrease in investor fear.

To illustrate, consider how the unrestricted linear ARDL (p, q) model of Eq. (1) is reduced to the following nonlinear asymmetric conditional ARDL (Apergis and Gangopadhyay 2020):

2b $$\begin{aligned} \Delta LnZ_{1t} & = \alpha_{0} + \rho LnZ_{1t - 1} + a^{ + } LnWEI_{t - 1}^{ + } + a^{ - } LnWEI_{t - 1}^{ - } + b^{ + } LnVIX_{{\left( {t - 1} \right) - 1}}^{ + } \\ & \quad + \,b^{ - } LnVIX_{{\left( {t - 1} \right) - 1}}^{ - } + \mathop \sum \limits_{i = 1}^{p - 1} \alpha_{i} \Delta LnZ_{1t - i} + \mathop \sum \limits_{i = 0}^{q - 1} ( b_{i}^{ + } \Delta LnVIX_{{\left( {t - 1} \right) - i}}^{ + } \\ & \quad + \,b_{i}^{ - } \Delta LnVIX_{{\left( {t - 1} \right) - i}}^{ - } ) + \mathop \sum \limits_{i = 0}^{q - 1} ( a_{i}^{ + } \Delta LnWEI_{t - i}^{ + } + b_{i}^{ - } \Delta LnWEI_{t - i}^{ - } ) + \omega_{t} \\ \end{aligned}$$

where $\mathop \sum \nolimits_{i = 0}^{q - 1} b_{i}^{ + }$ and $\mathop \sum \nolimits_{i = 0}^{q - 1} b_{i}^{ - }$ denote the short-run asymmetric dynamics of $LnVIX_{{\left( {t - 1} \right) - 1}}$, and $\omega_{t}$ labels the error term. The effects of positive and negative changes in $LnVIX_{{\left( {t - 1} \right) - 1}}$, and the asymmetric long-run coefficients of $LnVIX_{{\left( {t - 1} \right) - 1}}$, are calculated as $\theta^{ + } = - \frac{{b^{ + } }}{\rho }$ and $\theta^{ - } = - \frac{{b^{ - } }}{\rho }$.

$\mathop \sum \nolimits_{i = 0}^{q - 1} a_{i}^{ + }$ and $\mathop \sum \nolimits_{i = 0}^{q - 1} a_{i}^{ - }$ denote the short-run asymmetric dynamics of $\Delta LnWEI_{t - 1}$. The sums of positive and negative changes in *LnWEI*_*t*−1_ and the asymmetric long-run coefficients of $LnWEAI$ are calculated as $\beta^{ + } = - \frac{{a^{ + } }}{\rho }$ and $\beta^{ - } = - \frac{{a^{ - } }}{\rho }$. Now, the error correction model of Eq. (2b) can be presented as:

2c $$\begin{aligned} \Delta LnZ_{1t} & = \mathop \sum \limits_{i = 1}^{p - 1} \alpha_{i} \Delta LnZ_{1t - i} + \mathop \sum \limits_{i = 0}^{q - 1} ( b_{i}^{ + } \Delta LnVIX_{{\left( {t - 1} \right) - i}}^{ + } + b_{i}^{ - } \Delta LnVIX_{{\left( {t - 1} \right) - i}}^{ - } ) \\ & \quad + \mathop \sum \limits_{i = 0}^{q - 1} ( a_{i}^{ + } \Delta LnWEI_{t - i}^{ + } + b_{i}^{ - } \Delta LnWEI_{t - i}^{ - } ) +_{i} ECT_{t - 1} + \omega_{t} \\ \end{aligned}$$

#### Quantile autoregressive distributed lag model: fluctuations cointegrating relationships across quantiles

Following Cho et al. (2015), we proposed the QARDL model for the postulated ARDL model of (1) as follows:

3a $$\begin{aligned} Q\left( {\Delta LnZ_{1t} } \right) & = \sigma_{0} \left( v \right) + \mathop \sum \limits_{i = 1}^{p - 1} \delta_{i} \left( v \right)\Delta LnZ_{1t - 1} + \mathop \sum \limits_{i = 0}^{q1 - 1} \alpha_{i} \left( v \right)\Delta VIX_{{\left( {t - 1} \right) - 1}} \\ & \quad + \mathop \sum \limits_{i = 0}^{q2 - 1} \beta_{i} \left( v \right)\Delta WEI_{t - 1} + e_{t} \left( v \right) \\ \end{aligned}$$

In Eq. (3a), *e*_*t*_(*v*) = *Q*(*ΔLnZ*_1*t*_) − *ΔLnZ*_1*t*−*i*_(*v/F*_*t*−1_) and *ΔLnZ*_1*t*−*i*_(*v/F*_*t*−1_) is the *v*_*th*_ quantile of *ΔLnZ*_1*t*_, and it is dependent on the F_t−1_ information set.^7^ Now, incorporating the long-run dynamics into (1) the QARDL model can be written as follows:

3b $$\begin{aligned} & Q\left( {\Delta LnZ_{1t} } \right) = \sigma_{0} \left( v \right) + \pi \left( v \right)LnZ_{1t - 1} + \rho_{VIX} \left( v \right)LnVIX_{{\left( {t - 1} \right) - 1}} + \omega_{WEI} \left( v \right)LnWEI_{t - 1} \\ & \quad + \mathop \sum \limits_{i = 1}^{p - 1} \delta_{i} \left( v \right) \, \Delta \, LnZ_{1t - 1} + \mathop \sum \limits_{i = 0}^{q1 - 1} \alpha_{i} \left( v \right)\Delta LnVIX_{{\left( {t - 1} \right) - 1}} + \mathop \sum \limits_{i = 0}^{q2 - 1} \beta_{i} \left( v \right)\Delta LnWEI_{t - 1} + \, e_{t} \left( v \right) \\ \end{aligned}$$

Equation (3b), known as the QARDL error correction model (hereafter QARDL-ECM), posits the parameters of the short-run dynamics for food sales (*LnZ*_1_), *LnVIX*, and *LnWEI*, which are captured by *δ*_*i*_(*v*)*, α*_*i*_(*v*), and *β*_*i*_(*v*), respectively. Moreover, the corresponding long-run parameters are given by *ρ*_*VIX*_(*v*)*, ω*_*WEI*_(*v*). The cumulative short-run impacts of the independent variables can be calculated using the delta method: *α*_i*_(*v*) = $\mathop \sum \nolimits_{i = 0}^{q1 - 1} \alpha_{i}$(*v*)*, β*_i*_(*v*) = $\mathop \sum \nolimits_{i = 0}^{q2 - 1} \beta_{i}$(*v*)*.* Moreover, the long-run cointegrating coefficients can be assessed as *ρ*_VIX*_(*v*) = *ρ*_*VIX*_*/π, ω*_WEI*_(*v*) = *ω*_*WEI*_*/π.*

The convergence speed toward equilibrium, which must be negative and significant, is given as π*(*v*)*.*

Following Cho et al. (2015), we used the Wald test to statistically assess the regressors’ short- and long-term nonlinear and asymmetric effects on the regressand (*LnZ*_1_). In this context, the null and alternative hypotheses for short-term dynamics are as follows:

- *H*_0_*: S α*_*i*_(*v*) = *s* against *H*_1_*: S α*_*i*_(*v*) ≠ *s;*
- *H*_0_*: S β*_*i*_(*v*) = *s* against *H*_1_*: S β*_*i*_(*v*) ≠ *s;.*

For the cumulative short-run effect, the null and alternative hypotheses can be.

- *H*_0_*: S α*_i*_(*v*) = *s* against *H*_1_*: S α*_i*_(*v*) ≠ *s;*
- *H*_0_*: S β*_i*_(*v*) = *s* against *H*_1_*: S β*_i*_(*v*) ≠ *s;*

Meanwhile, for the long-run parameters and QARDL error correction term (ECT) terms (*π*(*v*)), the null and alternative hypothesis are as follows: *ρ*_*lnVIX*_(*v*)*, ω*_*lnWEI*_(*v*),

- *H*_0_*: S ρ*_lnVIX*_(*v*) = *S* (*ρ*_*lnVIXi*_*/π*) = *s* against *H*_1_*:S ρ*_lnVIX*_(*v*) = *S* (*ρ*_*lnVIXi*_*/π*) ≠ *s;*
- *H*_0_*: S ω*_lnWEI*_(*v*) = *S* (*ω*_*lnWEI*_*/π*) = *s* against *H*_1_*: S ω*_lnWEI*_(*v*) = *S* (*ω*_*lnWEI*_*/π*) ≠ *s;*
- *H*_0_: F π_*_(*v*) = *f* against *H*_1_: F π_*_(*v*) ≠ *f*.

Here, ***S*** and ***s***, and ***F*** and ***f*** are pre-determined matrices with the ***h*** number of restrictions. The Wald test examined all of the nonlinear features of the parameters. The relevant null and alternative hypotheses for the speed of adjustment parameter π_*_(*v*), for example, are:

- *H*_0_: π_*_(*0.25*) = π_*_(*0.50*) = π_*_(*0.75*) = π_*_(*0.95*) and
- *H*_1_: π_*_(*0.25*) ≠ π_*_(*0.50*) ≠ π_*_(*0.75*) ≠ π_*_(*0.95*) over the four quantiles Q25, Q50, Q75, and Q95.

As the independent variable moves from one quantile to another, QARDL allows us to establish a possible nonlinearity through a data-driven process. This method may be superior to Shin et al.’s (2014) nonlinear ARDL (NARDL), which exogenously builds nonlinearity by setting the quantile threshold to zero (see Apergis and Gangopadhyay 2020). Once (3b) is estimated, we can apply the Wald test to test the asymmetry hypothesis.

#### Endogeneity problems for the proposed models and insights from the Bewley transformation: a robustness check

The problem of endogeneity has been noted in the context of ARDL. A simple transformation of the ARDL model known as the Bewley transformation allows for asymptotically valid inference while overcoming estimation issues due to endogeneity using t-statistics on long-run coefficients. This transformation provides an alternative method for estimating the cointegrating relationship, with certain finite sample advantages over the Engel–Granger method. More information in the regression (in this case, via the Bewley transformation) is likely to produce estimators with better features, as simulations in Inder (1993) demonstrate.

By considering the ARDL (p, q) model from Eq. (4a), the final form of the same equation under the Bewley transformation can be written as Eq. (4b).

4a $$Y_{t} = \alpha_{0} + \mathop \sum \limits_{j = 0}^{q} \beta_{j} L^{j} X_{t} + \mathop \sum \limits_{i = 1}^{p} \gamma_{i} L^{i} Y_{t} + \varepsilon_{t}$$

4b $$Y_{t} = \frac{{\alpha_{0} }}{{1 - \gamma_{1} }} + \frac{{\beta_{0} - \beta_{1} }}{{1 - \gamma_{1} }}X_{t} - \frac{{\beta_{1} }}{{1 - \gamma_{1} }}\Delta X_{t} - \frac{{\gamma_{1} }}{{1 - \gamma_{1} }}\Delta Y_{t} + \frac{{\varepsilon_{t} }}{{1 - \gamma_{1} }}$$

Because of the presence of a contemporaneous relationship between the variables in Eq. (4b), the Bewley transformation necessitates the use of instrumental variables. Generally, $Y_{t - 1}$ is used as an instrument for $\Delta Y_{t}$. In this study, we will use Stata’s IVREG2 command to extract the Bewley transformation results and test the tenability of our ARDL model.

## Results and discussion

Table 7 illustrates the descriptive statistics of the data: the mean, median, and standard deviation of *LnZ*_1_. The kurtosis statistics of all series, except *WEI*, are greater than 3. Thus, the NARDL and QARDL models are more suitable. The Jarque–Bera tests find that all of the variables deviated from the normal distribution. Table 8 shows that all variables are I(0) or I(1), implying that the ARDL, NARDL, and QARDL models constitute the proper methodology. Tables 1, 2, 3, 4, 5 and 6 show the results of linear ARDL, NARDL, and QARDL model estimation.

**Table 1:** ARDL results

Variable	Dependent variable ***LnZ***_**1**_			
	*Long Run*		*Short Run*	
	Coeff	t-value	Coeff	t-value
*ECT*			− 1.03***	− 7.19
*LnVIX*	0.12***	4.76		
*LnWEI*	− 0.002	− 0.51		
Constant	22.98***	6.38		
*∆LnZ*_1*t*−1_			0.33***	2.90
R^2^	0.46		0.50	
F Stat	12.18***			
Cointegration	Yes			

Notes: *** indicate significant at 1% level of significance

**Table 2:** NARDL results

NARDL Model: LnZ1 = f (LnVIX, LnWEI)	
C	36.659***
*LnZ1*_*t*−1_	− 1.576***
*LnVIX*_*t*−1_^*POS*^	0.436***
*LnVIX*_*t*−1_^*NEG*^	0.134**
*WEI*_*t*−1_^*POS*^	− 0.029***
*WEI*_*t*−1_^*NEG*^	0.030***
*ΔLnZ1*_*t*−1_	0.403**
*ΔLnVIX*_*t*_^*POS*^	0.053
*ΔLnVIX*_*t*−1_^*POS*^	− 0.342***
*ΔLnVIX*_*t*_^*NEG*^	0.135*
*ΔLnVIX*_*t*−1_^*NEG*^	0.178**
*ΔWEI*_*t*_^*POS*^	0.025
*ΔWEI*_*t*−1_^*POS*^	− 0.003
*ΔWEI*_*t*_^*NEG*^	− 0.013
*ΔWEI*_*t*−1_^*NEG*^	0.035**
*F_PSS*	18.2115***
*t_BDM*	− 6.8854***
**Cointegration**	**Yes**

*Long-run effect*	
*L*_*LnVIX*_^*POS*^	0.276***
*L*_*LnVIX*_^*NEG*^	− 0.085**
*L*_*WEI*_^*POS*^	− 0.018***
*L*_*WEI*_^*NEG*^	− 0.019***

*Asymmetry effect*			
*Long-run asymmetry F-stat*		*Short-run asymmetry F-stat*	
*LnVIX*	37.75***	*LnVIX*	13.01***
*WEI*	35.80***	*LnWEI*	0.0000133
J-B test		0.851	
Ramssey RESET test		0.621	
Portmanteay test		13.380	
Breusch-Pegan test		0.054	

Notes: ***, **, and * indicate significant at 1%, 5%, and 10% significance levels

**Table 3:** QARDL results

Parameters	Quantile v = 0.25		Quantile v = 0.50		Quantile v = 0.75		Quantile v = 0.95	
	Coef	*p* value	Coef	*p* value	Coef	*p* value	Coef	*p* value
π_*_ (v)	***− 1.062***^***a***^	***0.000***	***− 0.961***^***a***^	***0.002***	***− 0.919***^***b***^	***0.042***	***− 1.250***^***b***^	***0.044***
ρ_VIX*_ (v)	0.060	0.429	0.100	0.150	***0.363***^***a***^	***0.000***	***0.254***^***a***^	***0.002***
ω_WEI*_ (v)	− 0.004	0.190	− 0.005	0.329	***0.013***^***b***^	***0.041***	0.009	0.175
ꝺ $\delta_{1}$(v)	0.183	0.452	0.109	0.712	0.120	0.660	0.318	0.417
ꝺ $\alpha_{0}$(v)	− 0.022	0.649	0.009	0.860	0.121	0.216	***0.181***^***c***^	***0.096***
ꝺ $\alpha_{1}$(v)	0.008	0.923	− 0.012	0.885	− 0.150	0.144	− 0.082	0.494
ꝺ $\beta_{0}$(v)	− 0.005	0.839	− 0.005	0.864	0.018	0.480	0.033	0.211
ꝺ $\beta_{1}$(v)	0.013	0.552	− 0.001	0.956	0.023	0.251	− 0.035	0.288
ꝺ $\beta_{2}$ (v)	− 0.009	0.661	0.008	0.654	0.021	0.184	***0.043***^***a***^	***0.007***
σ_*_(v)	***24.593***^***a***^	***0.000***	***22.149***^***a***^	***0.002***	***20.503***^***b***^	***0.044***	***28.339***^***b***^	***0.047***

Notes: The coefficients in bold-italics with a, b, and c indicates level of significance at 1%, 5%, and 10%, respectively

**Table 4:** Wald test results

Parameters	Coefficient	F-Stat
π_*_ (v)	0.24	0.8685
ρ_VIX*_ (v)	***8.45***^***b***^	***0.0374***
ω_WEI*_ (v)	5.56	0.1353
ꝺ $\delta_{1}$(v)	0.40	0.7558
ꝺ $\alpha_{0}$(v)	1.12	0.3516
ꝺ $\alpha_{1}$(v)	0.56	0.6420
ꝺ $\beta_{0}$(v)	0.63	0.5966
ꝺ $\beta_{1}$(v)	***4.11***^***b***^	***0.0112***
ꝺ $\beta_{2}$ (v)	1.76	0.1687
*Short-run Cumulative Effect*		
ꝺ $\alpha_{*}$	0.96	0.8115
ꝺ $\beta_{*}$	3.16	0.3673

Notes: The coefficients in bold-italics with a, b, and c indicates level of significance at 1%, 5%, and 10%, respectively

**Table 5:** Results from first stage regressions of Bewley transformation

$\Delta LnZ_{1t - 1}$	Coefficient	*p* value
$LnZ_{1t - 1}$	− 0.772	0.000
$LnVIX$	0.120	0.000
$WEI$	0.001	0.617
Cons	17.682	0.000
Sanderson-Windmeijer multivariate F test of excluded instruments: F (1, 51) = 34.17, Prob > F = 0.0000		
Under identification Test: Anderson canon. corr. LM statistic: Chi-sq(1) = 22.07, *p* value = 0.0000		
Weak identification Test: Cragg-Donald Wald F statistic 34.17***		
Weak-instrument-robust inference Anderson-Rubin Wald test F(1,51) = 2.97 *p* value = 0.0907 Anderson-Rubin Wald test Chi-sq(1) = 3.21 * p* value = 0.0733 Stock-Wright LM S statistic Chi-sq(1) = 3.03 * p* value = 0.0817		
Number of observations N = 55 Number of regressors K = 4 Number of endogenous regressors K_1_ = 1 Number of instruments L = 4 Number of excluded instruments L_1_ = 1		

Notes: *** indicate significant at 1% level of significance

**Table 6:** Results of Bewley transformation from instrument variable estimation

$LnZ1$	Coefficient	*p* value
$\Delta LnZ_{1}$	− 0.295	0.167
$LnVIX$	0.156	0.000
$WEI$	0.002	0.612
Cons	22.899	0.000
Number of obs = 55 F(3, 51) = 7.89 Prob > F = 0.0002 Total (centered) SS = 0.3189746795 Total (uncentered) SS = 30,134.53036 Residual SS = 0.3008455664 Centered R2 = 0.0568 Uncentered R2 = 1.0000 Root MSE = 0.07396		
Under identification test (Anderson canon. corr. LM statistic): 22.065 Chi-sq(1) * p* value = 0.0000 Weak identification test (Cragg-Donald Wald F statistic): 34.168 Stock-Yogo weak ID test critical values: 10% maximal IV size 16.38 15% maximal IV size 8.96 20% maximal IV size 6.66 25% maximal IV size 5.53 Sargan statistic (overidentification test of all instruments): 0.000 (Equation exactly identified)		

In Table 7, we explore the fundamental statistics before moving on to the unit root tests, summarized in Table 8. Tables 7 and 8 are available in the appendix. We use the ARDL methodology to explore the cointegrating relationship between variables given by Eq. (1), and the results are summarized in Table 1.

**Fig. 1:**
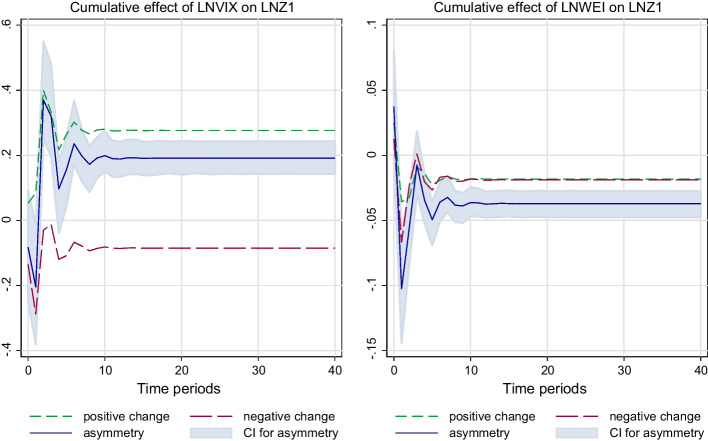
Cumulative effects of *LNVIX* and *WEI* on Food Insecurity (*LNZ*_1_)

The NARDL methodology is then applied, and the results are presented in Table 2. Figure 1 depicts the NARDL diagram.

In addition to the preceding tests, we use the QARDL methodology as a robustness check for the ARDL and NARDL methodologies. The possibility of the cointegrating relationship fluctuating across quantiles is central to the QARDL methodology. If this relationship fluctuates, the ARDL and NARDL results are not reliable. Table 3 displays the results of the QARDL model. Meanwhile, Table 4 displays the Wald statistics for the QARDL model.

The heterogeneity of coefficients across different quantiles is captured in Fig. 1, as we note that the relationship between *Food Security* (*LnZ*_1_) vis-à-vis *LnVIX* and *WEI* fluctuates from quantiles to quantiles.

We discuss the QARDL model results in light of the ARDL and NARDL results presented in Section "Results and discussion". Finally, one of the shortcomings of using autoregressive methodology is addressed by employing the Bewley transformation to examine endogeneity issues. We present the results from the Bewley transformation in Section "Findings from the Bewley transformation after controlling for endogeneity".

### Findings from the Baseline ARDL Model

The ARDL results (Table 1) reveal a long-term causality running from the chosen variables (*WEI* and *VIX*) to food sales (*Z*_1_), as the ECT, which indicates the speed of adjustment toward the long-run equilibrium after a shock, is negative and statistically significant at the 1% level. However, there is some evidence of overcorrection (ECT = *π* =  − 1.03). Second, we find that the F statistic confirms cointegration between food sales and the regressors at the 1% significance level for the ARDL model. Third, none of the control variables have long-term relationships with food sales: we find clear evidence that *VIX* positively impacts food sales (*Z*_1_) at the 1% significance level. However, the effect of *VIX* on *Z*_1_ is inelastic: a 1% increase (decrease) in *VIX*, or a 1% increase (decrease) in investor fear, increases (decreases) food sales (*Z*_1_) by 0.12%. Moreover, *WEI* did not exert any statistically significant long-term impact on food sales (*Z*_1_). In the short-run, we find that *∆LnZ*_1*t*−1_ has a statistically significant and positive impact on *∆LnZ*_1*t*,_ though inelastic, implying some sort of buyers’ panic to hoard food items: a 1% increase in *∆Z*_1*t*−1_ increases *∆Z*_1*t*_ by 0.34% and the effect is statistically significant at the 1% level. Finally, the proposed model is confirmed to have a long-run relationship, or cointegration, as evidenced by the bound test results provided by actual values of F_PSS in Table 1. As previously discussed, the model establishes causality running from the model’s explanatory variables to food sales, as evidenced by the error correction terms in Table 1.

Since the ARDL model assumes symmetric changes, it cannot detect asymmetric effects and, as a result, cannot detect hidden cointegration and nonlinearities. If there is hidden cointegration between food sales and the *VIX* and *WEI*, the ARDL model results are not credible. In what follows, we present the NARDL model results and find evidence of hidden cointegration, confirming that the baseline ARDL model results are not tenable.

### Findings from the NARDL model: is there evidence of hidden cointegration?

Table 2 shows that the positive partial sum of an increase in *LnVIX* ($\theta^{ + }$) has a positive long-term effect on *LnZ*_1_ and is statistically significant at the 1% level. The effect is inelastic, implying a 1% increase in *VIX*—*ceteris paribus*—will increase *Z*_1_ by 0.27%. Furthermore, the negative partial sum of a decrease in *VIX* ($\theta^{ - }$) has a long-term negative effect on *LnZ*_1_ and is statistically significant at the 1% level. The effect is inelastic once more. We find that the value implies that a 1% decrease in *VIX* increases demand for food sales (*Z*_1_) by 0.085%. Thus, there is evidence of asymmetry for *VIX*’s effect on *Z*_1_. In other words, there is evidence of hidden cointegration that the ARDL model failed to detect. However, the absolute values of the positive effect, $\theta^{ + }$, is stronger than the corresponding values of the negative effects, or $\theta^{ - }$. The positive effect is approximately three (3) times greater than the negative effect, in absolute value. Thus, the effect of *VIX* increases outweigh the effect of *VIX* decreases during the COVID-19 pandemic. The Wald test results (Table 4) confirm the long-term asymmetries of both effects of $\theta^{ + } \,{\text{and}}\, \theta^{ - }$.

Turning our attention to the asymmetric relationship between *Z*_1_ and *WEI*, we find that the positive partial sum of an increase in *WEI* ($\beta^{ + }$), has a negative long-term effect on food sales (*Z*_1_) and is statistically significant at the 1% level. The inelastic effect implies that a 1% increase in *WEI* will reduce food sales (*Z*_1_) by 0.018%. Moreover, the negative partial sum of a reduction in *WEI* ($\beta^{ - }$) has no statistically significant effect on *Z*_1_. In other words, there is significant asymmetry in the positive effect ($\beta^{ + }$) vis-à-vis the negative effect ($\beta^{ - }$) on food sales (*Z*_1_). The Wald test results (see Table 4) confirm the long-term asymmetries of both effects of $\beta^{ + }$ and $\beta^{ - }$.

For the short-term asymmetries, from the Wald test in Table 4, we find evidence of statistically significant asymmetries for *VIX*. As *VIX* rose by 1%, food sales (*Z*_1_) declined by 0.34%, at the 1% significance level. However, as *VIX* decreased by 1%, food sales (*Z*_1_) declined by 0.18%, at the 1% significance level. We did not find any evidence of short-run asymmetries for the effects of *WEI* on food sales (*Z*_1_).

The adequacy of the dynamic specification is first assessed using a variety of diagnostics, including the Jarque–Bera (J-B) statistic for error normality, the Portmanteau test statistic for model fit, the Breusch-Pagan test for autoregressive conditional heteroskedasticity up to order 2, and the Ramsey RESET statistics for regression specification error test. The results are shown in the lower panel of Table 4. The models pass key diagnostics, indicating error normality, the absence of autocorrelation and the ARCH effect, and overall parameter stability. Accordingly, the dynamics of COVID-19 indicators are sufficiently specified.

Finally, the postulated model is confirmed to have a long-run relationship, or cointegration, as evidenced by the bound test results provided by actual values of F_PSS in Table 2. As previously discussed, the model establishes causality running from the model’s explanatory variables to food sales, as indicated by the ECT in Table 2. The presence of hidden cointegration, as confirmed by the asymmetric effects of *VIX* and *WEI* on *LnZ*_1_, renders the ARDL findings untenable, while the NARDL model results are meaningful. In what follows, we apply the QARDL model to test the robustness of the NARDL model. One of the NARDL model’s central assumptions is that the coefficients remained stable across quantiles. The tenability of the NARDL model breaks down when coefficients fluctuate from one quantile to another. In the following subsection, the QARDL model will test the robustness of the NARDL model.

### Findings from the QARDL model to test fluctuations of coefficients across quantiles: robustness check of NARDL model

We estimated the model using four quantiles (Q25, Q50, Q75, and Q95). The speed of adjustment parameter π_*_ is not only negative but also significant across the four quantiles, indicating the presence of long-run cointegrating relationships. This demonstrates that long-run cointegrating relationships are valid with both basic ARDL estimation and the QARDL model.

The long-run coefficients of the *VIX* index [ρ_VIX*_ (v)] have significant positive impacts on food units sold [*Z*_1_] in the 0.95 quantile, whereas weekly economic activity [ω_WEI*_ (v)] has insignificant impact across the quantiles.

The short-run parameters show that, among the regressors, the lag difference of food sales [ꝺ $\delta_{1}$(v)], *VIX* [ꝺ $\alpha_{0}$(v)], and *WEI* [ꝺ $\beta_{2}$ (v)] exhibits a significant positive effect on food sales only in the fourth quantile [v = 0.95].

**Fig. 2:**
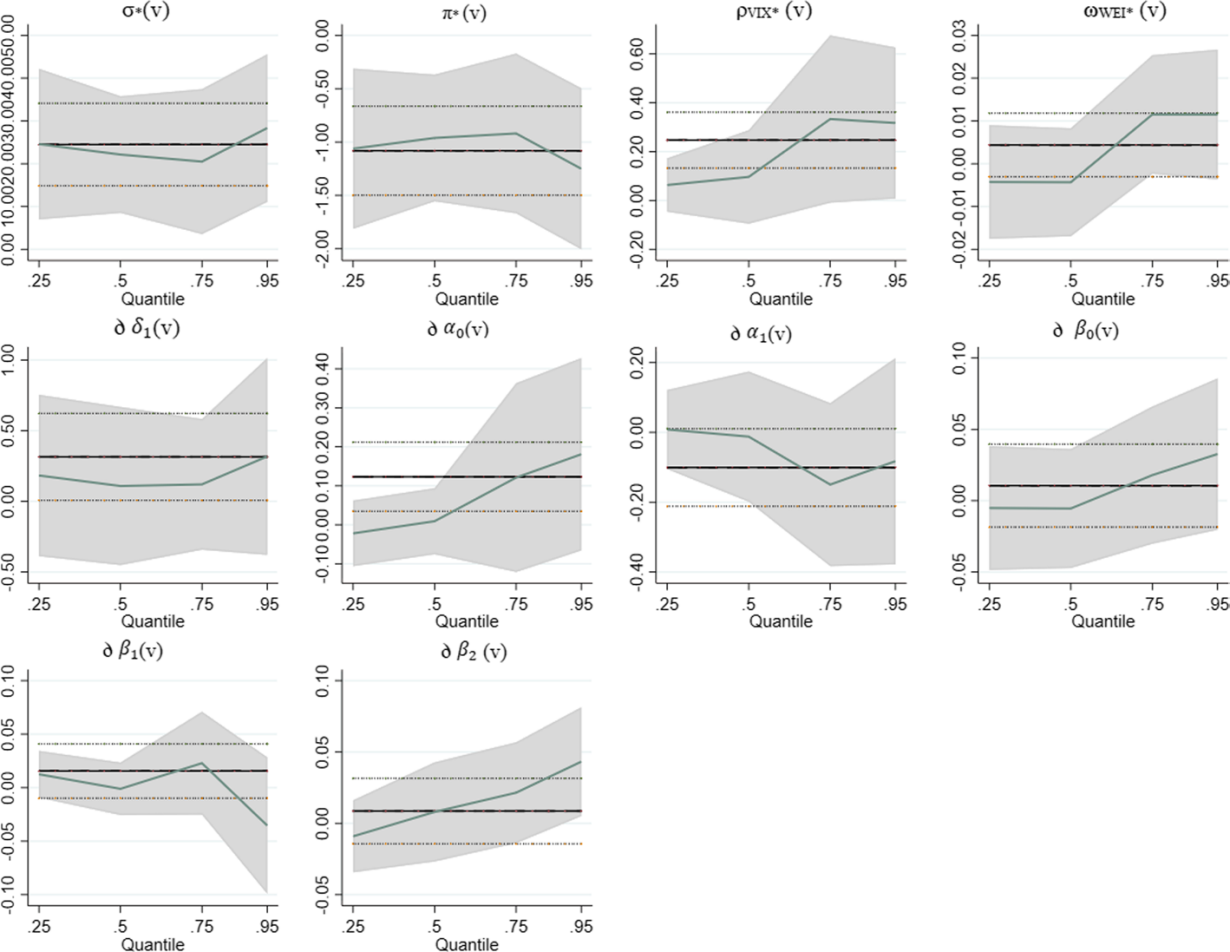
95% confidence intervals of the QARDL model parameters. Horizontal axis indicates the quantile levels (v < 0.5, 0.5 < v < 0.75, 0.75 < v < 0.95). Vertical axis indicates coefficient estimates of parameters

We discovered that the constant term [σ_*_(v)] is significant across all four quantiles. Figure 2 shows a graphical representation of the results.

Wald tests for null of linearity were performed to test for the consistency of parameter estimates across quantiles, and the results are shown in Table 3. For the speed of adjustment parameter π_*_, the *p* value of 0.418 shows that the null of linearity cannot be rejected, confirming that the parameter is consistent across quantiles. This conclusion is reached with the remaining parameters in both the long and short runs.

After applying the QARDL methodology for the same relationship between food sales and the control variables, we find important insights into the long-run relationships across various quantiles, that is, contingent on the conditional distribution of the dependent variables. It is important to note that the QARDL model findings do not support the NARDL model findings, but the Wald tests of the QARDL model show no evidence of parameter fluctuations or fluctuations in cointegration across quantiles. Thus, the NARDL model is robust.

Table 3 shows that the error correction terms across quantiles are negative and significant for all QARDL model quantiles. Hence, for all quantiles of the dependent variable, the causality runs from the chosen variables (*WEI* and *VIX*) to food sales (*Z*_1_). This is an important finding because both ARDL and NARDL results showed that causality runs from the selected variables to food sales at the 1% significance level. Thus, the unbundling of the relationship across quantiles—using the QARDL methodology—confirms the NARDL models’ causality findings for all quantiles.

### Findings from the Bewley transformation after controlling for endogeneity

It is critical to explain the causal identification strategy that we have chosen to address potential endogeneity in the current *WEI* and *VIX* measures. First, we avoided concurrent movements in *WEI* and *VIX* by delaying the *VIX* variable by one week. As a result, unknown future *WEI* values do not affect current or historical *VIX* values. Second, we checked the correlation between *WEI* and *VIX* (lagged by a year), and the correlation is in the safe zone. Finally, we check the reverse causality of *VIX* (expectations about the future) on current and future *WEI*. Then, we ran the ARDL estimation and found the absence of any long-run causality running from *VIX* to (future) *WEI*.

This subsection addresses the additional endogeneity problem by estimating the postulated model’s Bewley equation using the ARDL instrumental variable (IV). This will allow us to determine whether the proposed ARDL models are robust in extracting short and long-run relationships by using the lag of the dependent variable as the independent variable (Stučka 2004). The Bewley transformation allows us to find whether a change in the ARDL model’s dependent variable should correlate with any of the variables in question (see Inder 1993).

This transformation methodology uses the lagged dependent variable as the instrument. Tables 5 and 6 summarize the results of the instrumental variable regression using the IVREG2 command of Stata. Our findings are two-fold:

(i) As shown in Table 6, the Cragg–Donald Wald F statistic is greater than the Stock–Yogo weak ID test critical values. Hence, the instrument of the Bewley transformation is valid.
(ii) *Z*_1_ bears a (statistically significant) positive relationship with *VIX* (Table 6). Thus, increases (decreases) in the financial uncertainty, or disruptions, will increase (decrease) food sales (*Z*_1_). We find a positive relationship between *Z*_1_ and *WEI*, but it is not statistically significant. Note that the relationship between *WEI* and *Z*_1_ is expected to hold because these are concurrent movements within the same week. *WEI* is likely to take a little longer to influence food sales *Z*_1_. Thus, once the Bewley transformation controls the potential endogeneity, there is clear evidence that financial market disruptions or the *VIX* impacts food sales. After controlling for endogeneity, we determined that economic disruptions have no statistically significant effect on food sales in the same week. The standard ARDL results confirmed this. Hence, our identification strategy to lag *VIX* effectively avoids the endogeneity trap.

### Direct vis-à-vis indirect effects of the pandemic on food sales

One of our analysis’s obvious flaws is that the direct effect of the pandemic on food sales cannot be clinically separated from the indirect effect of the pandemic due to economic dislocations.^8^ We tested the addition of a variable to capture pandemic intensity as an additional regressor. However, as highlighted in the literature, any attempt to capture the intensity of the pandemic will create serious estimation problems. Our model’s apparent weakness stems from one of the primary requirements of any variant of the ARDL methodology: the regressors are truly exogenous (see Cho et al. 2021, p. 1). It is well-recognized that a large number of studies have ignored this key requirement and drawn incorrect conclusions. Sam et al. (2019) succinctly argued that “However, as pointed out by McNown et al. (2018), these assumptions were sometimes ignored by researchers, possibly leading to misleading conclusions” (p. 130).

In all ARDL variants, including ARDL, NARDL, QARDL models, and other extensions, the explanatory variables must be exogenous; hence, we chose our model after carefully checking that the chosen explanatory variables are exogenous. To assess the feasibility of incorporating the pandemic variable, we chose a variable *X*_*Pandemic*_, defined as the weekly COVID-19 infection rate, as a possible explanatory variable to capture the intensity of the pandemic in the United States.

The results from a vector autoregressive (VAR) model establishes that *X*_*Pandemic*_ cannot be used simultaneously with *WEI* due to endogeneity problems, as highlighted by Cho et al. (2021). Table 9 in the appendix confirms that *WEI* and *VIX* can be used safely as exogenous explanatory variables. However, including *X*_*Pandemic*_ in the ARDL methodology will lead to incorrect estimation. Several other pandemic-related variables were investigated. We found that none of them can be used with *WEI* and *VIX* without violating the requirement of exogenous explanatory variables.

However, the cost of this weakness in the ARDL methodology is relatively minor, if not non-existent. As shown in Table 10 in the appendix, *X*_*Pandemic*_ (from ARDL results after dropping *WEI*) has no cointegration with, or causal effect, on *Food Sales* (*Z*_1_) based on the bound test results. Hence, the results of our paper, which are based on several variants of the ARDL methodology, are robust because they have little weakness from the failure to separate the overall effect of the pandemic into direct and indirect effects (via *WEI*).

Therefore, we can say that the COVID-19 pandemic caused unprecedented shocks in global economic and financial markets. The collapse of economic and financial markets created downside risks. Because downside risks are a major concern in asset pricing and corporate finance, several important papers in finance have investigated this issue (see Wen et al. 2019). Traditionally, during an economic and financial turmoil, such as the COVID-19 pandemic, downside risk is measured by stock price crash risk. This risk, which will have long-term negative effects on the development of the capital market and economic growth, will jeopardize shareholder value. The problem is exacerbated by managers’ incentives to send biased signals to investors by withholding information (see Jin and Myers 2006). Firm-specific shocks do not emerge until a critical point and, more often than not, trigger a crash when the information becomes public (see, Habib et al. 2018; Callen and Fang 2013; Hutton et al. 2009).^9^ Note that the COVID-19 pandemic sent shockwaves through the entire economy, leaving little room for information asymmetry. Even if there are reasonable grounds to believe that the pandemic has created information asymmetry, retail investor attention can mitigate the problem of information withholding and asymmetry, as highlighted by Gao et al. (2018). According to Gao et al. (2018), retail investor attention can help overcome the problem of information withholding caused by asymmetric information because individual investors can glean information about a company from retail investor attention. Additional signals, such as retail investor attention, can help mitigate the asymmetric information problem (see Ding and Hou 2015). Consequently, the severity of crash becomes much less pronounced with retail investor attention.

The pandemic also caused significant changes in buyer behavior, particularly for food items, which can pose significant risks (see Li et al. 2022). Li et al. (2022) emphasized the emergence of market and financial risks due to changing buyer behavior. Li et al. (2022) have highlighted clustering in this context to better understand the role of unknown sub-patterns in data aggregation in local areas. Our focus on the US food market, rather than the global market or aggregate consumer spending, stems from the need for data aggregation in local areas.

During the pandemic, decision-making problems have become complex processes even for households. As Kou et al. (2021) highlighted, there is a need to develop more accurate and effective results for any complex decision-making problem. In this paper, we develop a quantile dependence structure as a more accurate model to explore food purchasing patterns across quantiles during unprecedented downside risks.

## Conclusion

From the baseline ARDL model, we find that the US food sales (*Z*_1_), during the first phase of the pandemic, had a long-run relationship (or cointegration) with both *VIX* and *WEI* and the causality runs from *VIX* and *WEI* to *Z*_1_. The baseline ARDL model suggests that *VIX* impacted *Z*_1_; however, there is no evidence that *WEI* had any statistically significant effect on *Z*_1_. To avoid the potential endogeneity between *VIX* and *WEI*, we have used the lagged value of *VIX* by a week in the regressions. We also used the Bewley transformation to overcome endogeneity problems. As a result, economic disruptions caused by mitigation strategies did not reduce US food consumption on its own. We find that investor apprehension, or fear, in financial markets (*VIX*), spilled over to US food market sales: *ceteris paribus*, a 1% increase in *VIX* (rising investor anxiety) increased weekly food sales by 0.12% in the long-run. The ARDL methodology assumes that this effect is symmetric: a 1% decrease in *VIX* reduces weekly food sales by 0.12%. The Bewley transformation estimation confirmed the ARDL findings.

As a robustness check, we used the nonlinear ARDL (NARDL) methodology to test the ARDL findings’ robustness by capturing the asymmetric effects of *WEI* and *VIX* on *Z*_1_. The NARDL results revealed a *hidden cointegration*, or long-run relationship, between *Z*_1_ and changes in *WEI* and *VIX*. Hence, the ARDL model results are found to be untenable. Using the NARDL methodology, we discovered significant long-run and short-run asymmetries: increases in *WEI* have adverse long-run effect on food sales, which is statistically significant at the 1% level. However, reductions in *WEI* had no statistically significant (long-run) effect on food sales. Thus, there is no evidence of food insecurity in the United States due to COVID-19 economic disruptions. We found that a 1% increase in the *VIX*, indicating increased investor fear, increased food sales by 0.27% in the long-run. This rise, driven by rising investor fear, could be explained as speculative hoarding/panic buying by buyers. By contrast, a 1% drop in the *VIX* resulted in a 0.088% increase in (long-run) food sales. Thus, there is no evidence that any positive or negative shocks to financial markets have harmed food sales in the United States in the long-run. Although increases (decreases) in *VIX* reduced (increased) food sales in the short-run, fluctuations in *WEI* had no statistically significant (asymmetric) effects on food sales.

When applying the QARDL methodology for testing the tenability of NARDL results, we find that the cointegrating coefficients in the long-run relationship between food sales and regressors have some fluctuations across different quantiles. However, except for the top quantile, the effects are not statistically significant. Thus, the NARDL results are robust when we consider the possibility of fluctuations in the postulated cointegrating relationship across quantiles. Therefore, despite nontrivial COVID-19-induced economic and financial disruptions, we conclude that US food sales remained relatively immune to such massive economic and financial disruptions.

## Data Availability

The Datasets are available from the following sources: Food Sales (Z_1_): US Department of Agriculture (USDA); Weekly Economic Index (WEI): Federal Reserve Bank of New York; CBOE Volatility Index (VIX): Chicago Board Options Exchange.

## Appendix

See Tables 7, 8,9 and 10.

**Table 7:** Descriptive statistics

Tests	*Food sales* (*LnZ*_1_)	*LnVIX*	*LnWEI*
Mean	23.40402	3.308548	− 4.53375
Median	23.39508	3.276573	− 4.175
Maximum	23.7092	4.336637	2.01
Minimum	23.23164	2.558002	− 11.43
Std. Dev	0.079684	0.345731	3.885991
Skewness	1.376243	0.578612	− 0.07226
Kurtosis	7.683862	4.173201	2.082183

**Table 8:** Unit root tests

Variables	Intercept				Trend & intercept			
	I (0)		I (1)		I (0)		I (1)	
	t-value	Prob	t-value	Prob	t-value	Prob	t-value	Prob
*ADF Test Results*								
* LnZ*_1_	− 4.464664	0.0007	− 7.958035	0.0000	− 4.451689	0.0041	− 7.939127	0.0000
* LnVIX*	− 2.800662	0.0647	− 7.136504	0.0000	− 3.083342	0.1205	− 4.780847	0.0016
* LnWEI*	− 2.363666	0.1568	− 3.798901	0.0051	− 2.767857	0.2153	− 4.111416	0.0107
PP Test Results								
* LnZ*_1_	− 4.240205	0.0014	− 16.13667	0.0000	− 4.170083	0.0091	− 18.04663	0.0000
* LnVIX*	− 2.954826	0.0457	− 7.151691	0.0000	− 3.127007	0.1104	− 7.184270	0.0000
* LnWEI*	− 1.805652	0.3741	− 3.779356	0.0054	− 1.950793	0.6145	− 4.100376	0.0110

**Table 9:** Vector autoregressive model of *LnVIX* and *LnWEI* with X_Pandemic_ as an exogenous variable

Variable	Dependent variable			
	*LnWEI*		*LnVIX*	
	Coeff	t-value	Coeff	t-value
*X*_*Pandemic*_	− 5.07***	− 3.23	− 0.15	− 0.45
*LnVIX*	0.31	0.55	0.80***	6.59
*LnWEI*	− 0.41***	− 3.90	0.03	1.57
Constant	0.57	0.43	0.75**	2.61

Notes: *** indicate significant at 1% level of significance

**Table 10:** ARDL results with pandemic intensity (*X*_*Pandemic*_) as a regressor

Variable	Dependent variable ***LnZ***_**1**_			
	*Long run*		*Short run*	
	Coeff	t-value	Coeff	t-value
*ECT*			− 0.35*	− 1.62
*LnVIX*	− 0.14	− 1.03	0.07*	1.76
*X*_*Pandemic*_	1.42*	1.94	− 0.18	− 0.90
Constant			8.36	1.64
*∆LnZ*_1*t*−1_			− 0.53***	− 2.78
R^2^	0.73		0.73	
F Stat	3.28			
Cointegration	NO			

Notes: *** and * indicate significant at 1% and 10% significance levels

## Footnotes

1 We choose a simple indicator, or proxy, to measure the economic consequences of the pandemic mitigation strategy, or what is called lockdowns, during the initial phase of the outbreak of the pandemic in the US from January 2020 to January 2021: we use the US weekly economic index (*WEI*) as an indicator to gauge economic disruptions and supply shocks caused by the COVID-19 mitigation strategy in the United States. Further details are provided in Section "Variables and data".

2 To avoid the trap of endogeneity between *VIX* and *WEI*, we choose the lagged value of *VIX* by a week: thus, *VIX* is not impacted by the future change in weekly activity index. We also checked the correlations between *VIX* and *WEI* and also a basic ARDL to ensure that the possibility of reverse causality from *VIX* to *WEI* is low. See Section "Baseline ARDL model" of this paper.

3 Since the ARDL methodology assumes symmetric effects of changes in *WEI* and *VIX* on *Z*_*1*_, our ARDL model is unable to detect hidden cointegration—characterised by asymmetric relationships between *Z*_*1*_ vis-à-vis positive and negative shocks in *VIX* and *WEI*. To overcome this weakness of our ARDL model, we will apply the nonlinear ARDL (NARDL) methodology that can detect hidden cointegration arising from asymmetric effects of positive and negative shocks in *VIX* and *WEI* on *Z*_*1*_. See Section "Nonlinear autoregressive distributed lag model: an extension" for full details.

4 Both ARDL and NARDL methodologies have a common shortcoming since both methodologies examine the relationship between food sales (*Z*_*1*_) vis-à-vis *WEI* and *VIX* only at the conditional mean and, thereby, ignore an important possibility that food sales might bear fundamentally heterogeneous relationships with its determinants across different quantiles of the conditional distribution *Z*_*1*_. If the postulated relationship is meaningfully heterogeneous across quantiles, then our findings NARDL will be untenable (see Section "Quantile autoregressive distributed lag model: fluctuations cointegrating relationships across quantiles").

5 The major weakness of the ARDL estimation is the potential endogeneity created by the lagged dependent variables. We apply the Bewley transformation to assess the relationship between food sales and other variables—after controlling for potential endogeneity (see Section "Endogeneity problems for the proposed models and insights from the Bewley transformation: a robustness check").

6 More details about the index can be found at https://www.newyorkfed.org/research/policy/weekly-economic-index#/. As discussed before, *WEI* and *VIX* are not contemporaneous movements—*VIX* is lagged by a week to avoid endogeneity.

7 See Kim and White (2003).

8 We are grateful to one of our referees for raising this inseparability of direct and indirect effects.

9 The extant literature considered various factors responsible for the severity of stock price crash risk: as examples, opaque financial reports (Hutton et al. 2009; Kim and Zhang 2014), corporate tax avoidance (Kim et al. 2011b), CFO equity incentives (Kim et al. 2011a), analyst optimism (Xu et al. 2013), institutional investor stability (Callen and Fang 2013), corporate social responsibility (Kim et al. 2014), religion (Callen and Fang 2015), CEO overconfidence (Kim et al. 2016), and social trust (Li et al. 2017).
